# Elevated Dip Water Temperatures Do Not Increase Risk of Thermal Injury During Cast Application

**DOI:** 10.51894/001c.164361

**Published:** 2026-07-31

**Authors:** Andrew Prieskorn, Jonathan Avon, Daniel Prieskorn, David Prieskorn

**Affiliations:** 1 Pikeville College of Osteopathic Medicine

## 34

### Introduction

Nonoperative fracture management through casting reflects osteopathic principles emphasizing the body’s capacity for self-regulation and self-healing when structural alignment is preserved. Thermal burns have been reported as a complication of cast application and are often attributed to elevated dip water temperatures. These concerns may delay immobilization and compromise efficient patient care. The objective of this study was to determine whether elevated dip water temperatures produce skin temperatures capable of causing thermal injury during routine cast application.

### Methods

Sixty short arm casts were applied to a single healthy volunteer under standardized environmental conditions. Fiberglass and plaster casts were immersed in either cool (14°C) or warm (77°C) water prior to application. Both forearms were tested simultaneously, with one arm serving as a control. Three independent temperature-monitoring devices were placed directly on the skin beneath standardized padding, and infrared thermometry measured external cast temperatures. Temperatures were recorded for ten minutes following application. Peak temperatures and time to maximal temperature were analyzed using independent samples t-tests.

### Results

Across all groups, external cast temperatures remained below established thresholds for thermal injury and did not exceed 45°C in plaster casts or 51.8°C in fiberglass casts. Mean internal temperatures peaked at 32.1°C beneath fiberglass casts dipped in cool water and 40.9°C beneath plaster casts dipped in warm water (*p* < .001). No measurements approached levels associated with cutaneous damage. Warmer dip water temperatures reduced cast setting time by an average of two minutes. No skin injury was observed following any application.

### Conclusions

Elevated dip water temperatures, even at extreme levels, do not produce clinically meaningful thermal injury when standard casting techniques are employed in healthy limbs. These findings support osteopathic principles emphasizing nonoperative care and the body’s intrinsic capacity for healing when structural integrity and circulation are maintained. The safe use of warmer dip temperatures may improve efficiency and patient comfort without compromising tissue safety. Clinicians should continue to consider individual patient factors, including limb size, vascular status, and soft tissue injury, when applying casts. This study provides original in vivo evidence to guide safer, more effective conservative fracture management.

